# Delegation to automaticity: the driving force for cognitive evolution?

**DOI:** 10.3389/fnins.2014.00090

**Published:** 2014-04-29

**Authors:** J. M. Shine, R. Shine

**Affiliations:** ^1^Brain and Mind Research Institute, The University of SydneySydney, NSW, Australia; ^2^School of Biological Sciences, The University of SydneySydney, NSW, Australia

**Keywords:** automaticity, delegation, bipedality, evolution

## Abstract

The ability to delegate control over repetitive tasks from higher to lower neural centers may be a fundamental innovation in human cognition. Plausibly, the massive neurocomputational challenges associated with the mastery of balance during the evolution of bipedality in proto-humans provided a strong selective advantage to individuals with brains capable of efficiently transferring tasks in this way. Thus, the shift from quadrupedal to bipedal locomotion may have driven the rapid evolution of distinctive features of human neuronal functioning. We review recent studies of functional neuroanatomy that bear upon this hypothesis, and identify ways to test our ideas.

## Introduction

Identifying the adaptive significance of trait variation is a primary challenge for evolutionary biology, and few traits have attracted as much speculation as the wide gap in cognitive skills (as reflected in complex language, tool use, etc.) between *Homo sapiens* and all other extant taxa (Faure et al., 2005; Poldrack et al., 2005; Ramnani, 2006; Passingham, 2008; Doyon et al., 2009; D'Angelo and Casali, 2012). Notwithstanding increasing evidence of high-level cognition in non-humans (Doya, 2000; Mithen, 2005; Poldrack et al., 2005; Vanderschuren and Di Ciano, 2005; Graybiel, 2008; MacNeilage, 2008; Passingham, 2008; Doyon et al., 2009), no other taxon exhibits the same cognitive capacities as our own species. The fossil record reveals a rapid increase in brain size and complexity (plausibly reflecting an increase in cognitive ability) approximately 2.4 million years ago, soon after humans evolved another distinctive feature–bipedal locomotion (Alexander et al., 1986; Faure et al., 2005; Lewin, 2009; Ashby et al., 2010).

We suggest that bipedality directly favored a specific mechanism for neuronal functioning, which subsequently facilitated the rapid evolution of increased cognitive capacity in hominids. Our hypothesis differs from earlier speculations on the origins of human intelligence by focusing on a specific and distinctive aspect in which human cognition differs from that of other species (in degree if not in kind), and in identifying a potential selective advantage to the elaboration of that mechanism for neural functioning.

First, what is distinctive about the way in which the human brain functions, relative to other species? The large behavioral repertoire of humans leaves no doubt that the human brain differs from that of our closest evolutionary relatives (Smaers et al., 2012). However, comparative neurological studies demonstrate that the human brain does not contain any structures that are distinctly unique to humans. Rather, the brain has undergone expansion of pre-existing structures that have re-wired their connectivity (Mantini and Corbetta, 2013; Smaers and Soligo, 2013), leading to the creation of novel network architectures in the brain. Given this morphological conservatism, the distinctive features of the human brain are likely to involve the elaboration of pre-existing functions to facilitate increased behavioral complexity.

Most previous hypotheses about the evolution of distinctively human patterns of cognitive function have glossed over the nature of the differences, focusing instead on somewhat vague concepts like “greater intelligence.” That is, previous hypotheses have been predicated on the simplistic assumption that human brains somehow work “better” than the brains of our nearest relatives, at least when dealing with complex tasks, without identifying specific aspects either of brain function or of the morphological underpinnings of that function. Instead, they have focused on potential factors that allowed or favored an expansion in brain size relative to body size, and/or conferred a survival or reproductive advantage to individual proto-humans with “better” brains. These authors have identified many ecological, behavioral and physiological factors that may have favored increases in cognitive capacities in the human brain. Those factors include the expanding array of habitats and behavioral niches occupied by proto-humans (Laland et al., 2000), the advent of language (Häberling et al., 2010), increased levels of fatty acids in the diet (Crawford, 1992), increased group size (Laland et al., 2010), changes in climate (Ash and Gallup, 2007), and modified targets and intensity of sexual selection (Dunbar and Shultz, 2007a). However, many of these theories are difficult to falsify, because most identify a simple increase in “intelligence” or “cognitive ability” as the biological trait under selection.

To explain the large behavioral differences that exist between humans and our closest relatives, an effective theory of brain evolution should specifically consider both *what* has changed and *why* that change may have conferred an adaptive advantage to the individuals who exhibited the benefit. There is unlikely to be any simple answer to the question: “how do human brains function differently than those of our closest relatives?” Previous attempts to explain the distinctive behavioral capacities of humans have suggested that an increase in overall brain size relative to body size was the key evolutionary change. However, comparative analyses have questioned the validity of that putative “novel feature” of human brain size (Deaner et al., 2007; Dunbar and Shultz, 2007b). Other researchers have suggested that cortical expansion may have increased the information-processing capacities of the brain (Hill et al., 2010), or that the unique capacities of the human brain are achieved via an abundance of cortical architecture that can be utilized for abstract planning and processing (Buckner and Krienen, 2013). Although all of these factors may play a role, we have chosen to focus instead on a specific way in which our brains function, as it is the functioning of the brain that is exposed to the forces of natural selection.

We suggest that one of the most fundamental distinguishing characteristics of human cognition is the way in which the brain dynamically shifts the way in which it deals with repetitive tasks. When we first encounter a daunting task that requires complex responses to multiple inputs (say, driving a car, or learning to play a musical instrument), we concentrate on that task to the exclusion of almost everything else. Gradually, as we learn the appropriate motor responses to specific situations (presumably via an increase in rewarding feedback), we no longer need to devote our full attention to the performance of the task, and can instead focus on other issues. With increasing practice, the basic activities become “automatic,” freeing attentional resources that are only required when some unpredictable (or unusually challenging) situation arises.

Although the process of tasks becoming “fond nature” is familiar to all of us, scientific understanding of the mechanisms involved in this delegation of authority from one neural component to another is only just emerging (Alexander et al., 1986; Faure et al., 2005; Poldrack et al., 2005; Mithen, 2005; Ito, 2006; Graybiel, 2008; MacNeilage, 2008; Passingham, 2008; Doyon et al., 2009; Lewin, 2009; Balsters and Ramnani, 2011; Seger and Spiering, 2011). When a challenge is first encountered, it needs to be processed by a rapid and flexible system capable of analysing complex patterns and responding with a wide array of behavioral responses, each of which conveys its own precise probabilistic advantage for success (Alexander et al., 1986; Poldrack et al., 2005). However, once feedback has been received and the challenge has been mastered (to the point that the brain can accurately predict the optimal response to most situations that are likely to arise), the control of the task is then relegated to a “lower” level, outside of conscious control (Alexander et al., 1986; Hoshi et al., 2005; Poldrack et al., 2005; Doyon et al., 2009).

## The driving force behind human brain evolution

Although many species can transfer behavior from volitional to habitual function (Poldrack et al., 2005; Barton, 2007; Seger and Spiering, 2011; Krubitzer and Seelke, 2012; Barton and Venditti, 2013), the shift from quadrupedal to bipedal locomotion nonetheless may have been a powerful driver for the rapid elaboration of the distinctively human “delegation” mode of information processing. Bipedality is rare in mammals, seen commonly only in humans and in some apes (Hardman et al., 2002; Alexander, 2004; Doyon et al., 2009). Although bipedality plausibly affords a number of adaptive advantages (e.g., it facilitates surveillance in densely vegetated areas, and frees the arms for other tasks Carrier, 2011), it also imposes a massive information-processing challenge. Compared to the stability conferred by quadrupedal locomotion, a bipedal organism rests its body mass on only two support points. This inherently unstable posture means that even a tiny shift in position will cause a fall, unless the animal instantly detects and responds to that change. Presumably for this reason, quadrupedal animals that resort to bipedality for surveillance typically do so only briefly, or in highly stereotyped poses (as is the case with meerkats). Moving about while bipedal poses extraordinary challenges, whereby the individual must constantly respond to ever-changing subtle shifts in weight distribution (Preuschoft, 2004), reducing its ability to attend to other aspects of its environment (such as the detection of food sources or approaching predators).

Despite these challenges, adult humans spend little time consciously thinking about maintaining their balance as they move around, except when placed in a challenging circumstance, such as walking on a narrow beam or when leaving a pub. The means of achieving that liberation is very clear as one watches a young child learning to walk. This is a long process, with every step initially requiring full concentration. Through time, however, the skills develop as control over fine motor movements improves–and full concentration on movement is no longer needed as the tasks involved become “automatized” and are delegated to other parts of the brain, such as the basal ganglia (Poldrack et al., 2005; Ashby et al., 2010; Seger and Spiering, 2011; Sepulcre et al., 2012) and the cerebellum (Duncan, 2001; Desmurget and Turner, 2010; Balsters and Ramnani, 2011; Callu et al., 2013). Plausibly, then, the adoption of bipedalism in proto-humans posed a strong selective advantage for individuals with brains capable of using their full processing power to learn bipedalism, but that were also able to delegate the basic tasks of walking and running to “lower” neural centers, freeing up the higher segments for detecting unpredictable opportunities and challenges (be they related to predators, food, or social cues), and rapidly responding to that information.

In summary, we suggest that (1) the ability to delegate routine tasks from the cortex to other parts of the brain is more highly developed in humans than other species; and (2) that elaboration arose during our evolutionary history because the computational challenges associated with balancing on two legs enhanced individual fitness in proto-humans who were capable of transferring the control of routine tasks in this way. To this we can add (3) that once this “delegation” mode of neural functioning had evolved, it was co-opted for many other cognitive tasks–essentially, liberating the cortex to deal with novel unpredictable events.

Once a “delegation” system has evolved, the resultant circuitry can be co-opted for aspects of brain function other than simply motor movements. For example, humans probably deal with cognitive and emotional issues in much the same way (i.e., events that initially require full concentration, ultimately become processed subconsciously after frequent repetition Hikosaka et al., 1999; Lehericy et al., 2005; Mainzer, 2008; Hertel and Brozovich, 2010). Automatization also enables complex activities to be performed even during periods of extreme stress, when flexible cortical networks are overwhelmed but the cerebellum remains functional (Chandler et al., 2013). What parts of the human brain might fulfill these functions? Below, we review evidence as to the likely anatomical and functional basis for such a system.

## Mapping behavior to brain circuitry

Because a diverse suite of behaviors become automatic with practice, we doubt that these increases in behavioral capacity were driven by the evolutionary expansion of a single neural structure (Barton and Harvey, 2000; Weaver, 2005; Barton and Venditti, 2013; Smaers and Soligo, 2013). Instead, this flexibility was likely driven by the dimensional expansion of a pre-existing organizing principle for the brain. To achieve efficient automatization, such a neural system would require a unique set of traits. Early in the course of learning, the system would require ready access to moment-to-moment reinforcement signals. In addition, the behavioral capacities in the early stages should also be flexible, providing a large array of potential behaviors with which to learn the most effective strategy. Over the course of learning, this system should trade off flexibility for consistency, while also incorporating feedback in different ways, such as through small errors in action prediction rather than in the outcome of reinforcement trials (Frank, 2005; Hikosaka et al., 2008). Lastly, the system would require global access to all of the behavioral capacities of the brain, as there is now compelling evidence that automatization can occur in many non-motor functions, such as cognition (Graybiel, 1997) and perception (Bastian, 2011; Baumann and Mattingley, 2014), which utilize vastly different neural architectures.

**Figure 1:**
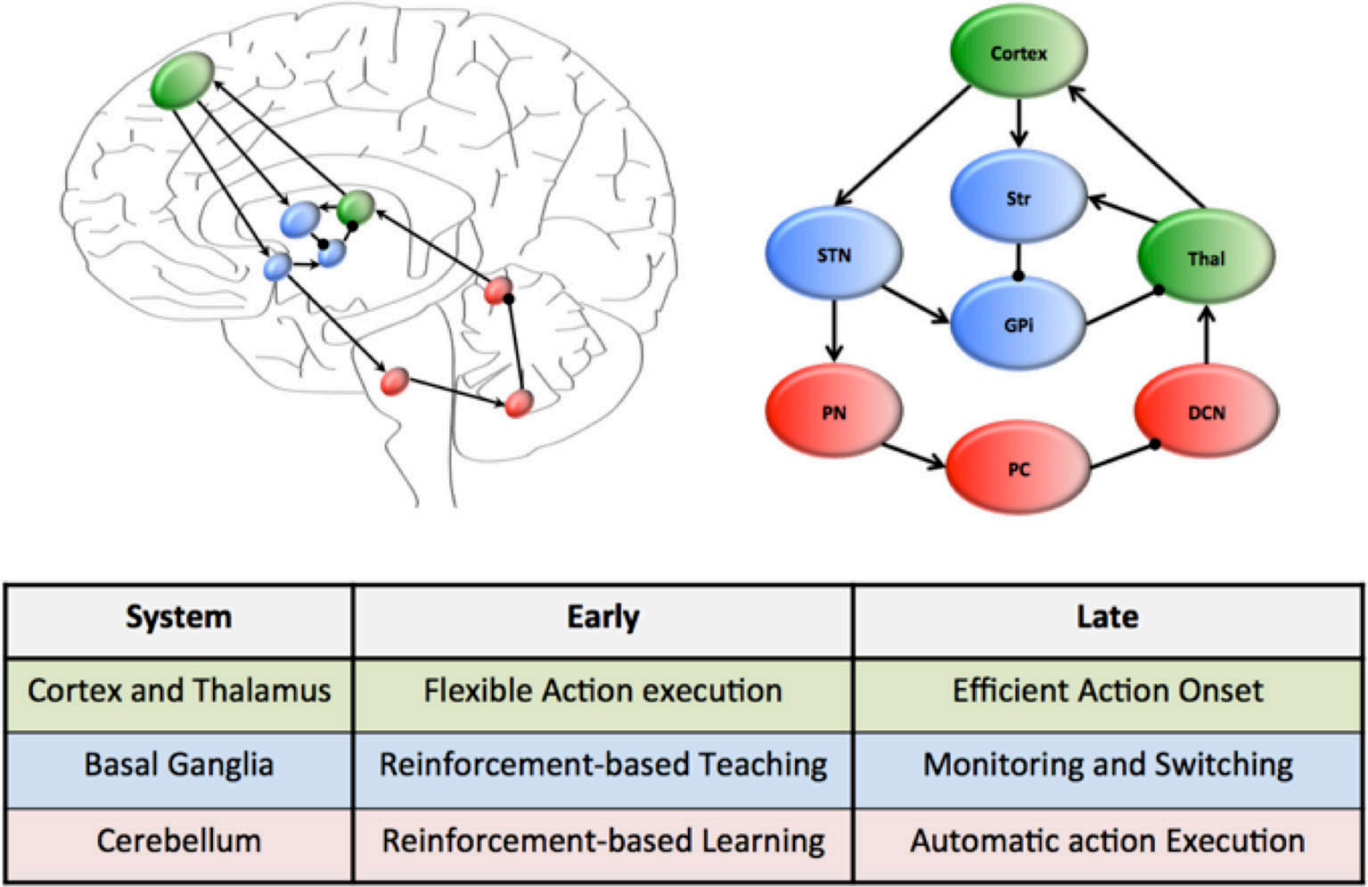
**Hierarchical neural circuits that control learning in the human brain**. The cortex (green) is preferentially involved early in the course of learning, allowing flexible responses to rapidly changing environmental contingencies. Through extensive connections with the basal ganglia nuclei (blue), which are under the regular influence of neuromodulatory signals reflecting prediction error and salience, the cortex is able to transfer the performance of complex tasks to rigid yet reproducible neuronal motifs within the cerebellar cortex, which is “trained” by the neuromodulatory signals and the basal ganglia to efficiently perform complex actions. The connectivity of this motif is organized along a rostrocaudal gradient, with rostral regions preferentially involved early in learning, and more caudal structures in tasks that require less cognitive control. The wiring diagram of this unit has been over-simplified to highlight the key circuitry responsible for communication within the system.

A plausible candidate system that simultaneously achieves each of these capacities is that of the cortico-cerebellar pathways (Weaver, 2005; Balsters et al., 2010; Smaers et al., 2011, 2013; Koziol et al., 2014). This system connects large regions of the cerebral cortex, which is characterized by flexible and rapid processing, with the cerebellum, which is responsible for habitual and inflexible processing (see Figure 1). In combination, the cerebral cortex and the cerebellum contain more than 70% of the neurons in the human brain (Shepherd, 2004) and are connected through a series of reciprocal loops, which themselves broadly map onto the functional capacities of the human brain (e.g., motor functions rely on caudal cortex and anterior cerebellum structures, whereas cognitive capacities rely on dorsolateral cortex and posterior cerebellum) (Figure 1) (Bostan et al., 2010, 2013).

Despite their connectivity with each other, the cerebral cortex and cerebellum are organized into quite different motifs of internal connectivity (Ito, 2006). For example, the cerebral cortex is a thin, multi-layered sheet with massive inter-connectivity across layers and regions (George and Hawkins, 2009), whereas the cerebellum consists of a network of simple cellular motifs, robustly repeated across the entire structure (Ramnani, 2006; D'Angelo and Casali, 2012). The structure of these motifs accords well with the notion that the cerebral cortex is primarily engaged early in unsupervised learning (when it is advantageous to respond flexibly to a novel stimulus) (Doya, 2000), yet decreases its activity over the course of learning, which may be due to increased neuronal efficiency (Ashby et al., 2007). In addition, it is also now clear that the cerebellum is important for the execution of automatized behaviors (Lang and Bastian, 2002; Balsters and Ramnani, 2011). Given the efficient neuronal architecture of the cerebellar cortex, we propose that the cerebellum plays a prominent role in the execution of learned behaviors, effectively liberating the more flexible architecture of the cortex to process novel behavioral challenges (Figure 1). Importantly, this mechanism can be mapped onto a functional corticocerebellar unit of the brain (Figure 1) which, depending on which neural and cerebellar regions are involved in the learning process, can effectively allow learning and automatization of motor, as well as cognitive and affective behavioral patterns (Graybiel, 1997, 2008; Hertel and Brozovich, 2010).

Despite convincing evidence that control over routine behaviors is gradually transferred from cortical to cerebellar circuitry (Faure et al., 2005; Ito, 2006; Ramnani, 2006; Passingham, 2008; Doyon et al., 2009; Balsters and Ramnani, 2011; D'Angelo and Casali, 2012; Balsters et al., 2013), little is known about the structures that mediate this transfer at the neural level. Such a system would require anatomical connectivity between the cerebral cortex and the cerebellum, along with adequate exposure to learning-related reward signals and signals related to environmental salience. The basal ganglia (a collection of highly conserved neural structures in the telencephalon) are ideally placed to fulfill these functions (Alexander et al., 1986; Doya, 2000; Faure et al., 2005; Hoshi et al., 2005; Mithen, 2005; Poldrack et al., 2005; Vanderschuren and Di Ciano, 2005; Graybiel, 2008; Passingham, 2008; Doyon et al., 2009; Lewin, 2009; Seger and Spiering, 2011). The striatum, a prominent member of the basal ganglia, is connected to the cerebral cortex and cerebellum in a gradient similar to that displayed by the cortico-cerebellar system (Alexander et al., 1986; Hardman et al., 2002; Alexander, 2004; Faure et al., 2005; Hoshi et al., 2005; McHaffie et al., 2005; Poldrack et al., 2005; Barton, 2007; Doyon et al., 2009; Lewin, 2009; Ashby et al., 2010; Seger and Spiering, 2011; Krubitzer and Seelke, 2012; Barton and Venditti, 2013) (Figure 1) and receives multiple subcortical and brainstem inputs reflecting reward prediction error (Alexander et al., 1986; Faure et al., 2005; Poldrack et al., 2005; Mithen, 2005; Ito, 2006; Graybiel, 2008; Kreitzer and Malenka, 2008; MacNeilage, 2008; Passingham, 2008; Doyon et al., 2009; Lewin, 2009; Balsters and Ramnani, 2011; Bromberg-Martin and Hikosaka, 2011; Seger and Spiering, 2011) and salience signals (Alexander et al., 1986; Poldrack et al., 2005; Bromberg-Martin et al., 2010). We suggest that early in the course of learning, the basal ganglia play an important role in training the cerebellum to perform effective action sequences through reinforcement of appropriate responses (Kawato et al., 2011). Once a behavior has become automatized, the role of the basal ganglia nuclei then shifts to a monitoring role, flexibly “switching” activity between these two systems in the face of changing environmental circumstances (Figure 1), a task to which it is also neuro-anatomically well suited (Aron et al., Saint-Cyr, 2003; Aron and Poldrack, 2006). The ability to efficiently and effectively “delegate” information to automaticity over the course of learning thus depends on the interplay between cortical and cerebellar neurons through their interconnections with the basal ganglia (see Figure 1).

We are not the first to suggest that the basal ganglia nuclei are integrally involved in automatization. Indeed, early researchers suggested that the basal ganglia may act as the neural substrate of motor learning, with more dorsal regions responsible for the initial learning of a behavior, and caudal regions becoming active during the consolidation of habitual responses (Alexander et al., 1986; Hoshi et al., 2005; Poldrack et al., 2005; Doyon et al., 2009; Ashby et al., 2010). However, recent evidence suggests that the basal ganglia are merely involved in the kinematic execution of motor patterns rather than in the storage of habits *per se* (Poldrack et al., 2005; Barton, 2007; Desmurget and Turner, 2010; Seger and Spiering, 2011; Krubitzer and Seelke, 2012; Barton and Venditti, 2013), aiding in the “selection” of appropriate actions based on environmental contingencies (Redgrave et al., 1999). In this manner, the geographical differences in basal ganglia involvement during motor learning can be explained by the response of separate regions of the basal ganglia to unique spatial frames of reference, with dorsal regions utilizing spatial reference and caudal regions utilizing motoric reference (Hikosaka et al., 1999; Hardman et al., 2002; Alexander, 2004; Lehericy et al., 2005; Doyon et al., 2009). Alternatively, the ventral-to-dorsal gradient within the striatum may reflect preferential involvement with goal-directed rather than habitual actions (Lehericy et al., 2005; Yin and Knowlton, 2006; Hikosaka and Isoda, 2010). In either case, the basal ganglia nuclei are likely to play an integral role in the transfer of information from cortical to cerebellar circuitry over the course of learning.

The modular architecture of these three inter-connected neural systems strongly supports the capacity for parallel processing in the brain (Balleine and Ostlund, 2007; Isoda and Hikosaka, 2011). Also, structural studies indicate that these three units are well conserved across the evolutionary history of mammals (Poldrack et al., 2005; Barton, 2007; Balsters et al., 2010; Bromberg-Martin and Hikosaka, 2011; Carrier, 2011; Seger and Spiering, 2011; Krubitzer and Seelke, 2012; Barton and Venditti, 2013; Buckner and Krienen, 2013). That conservatism is consistent with the idea that natural selection has elaborated a pre-existing system, by modifying its functionality (rather than, for example, by generating a novel structural component to the brain). Thus, the major mechanism behind human behavioral evolution may have been a shift in function rather than structure. That is, the massive increase in information processing required by bipedality conferred a selective advantage to responses at the neural level that accelerated an individual's capacity to “delegate” information processing from conscious, flexible control into more “automatic” systems that are rigid yet highly reproducible.

In keeping with this idea, anatomical studies have not discovered entirely novel brain regions unique to humans; instead, specific systems of the brain, including nuclei within the basal ganglia (Hardman et al., 2002; Alexander, 2004; Preuschoft, 2004; Doyon et al., 2009; Bromberg-Martin et al., 2010; Sepulcre et al., 2010) and connections between the anterior cerebral cortex and the lateral cerebellum (Poldrack et al., 2005; Ashby et al., 2010; Balsters et al., 2010; Carrier, 2011; Seger and Spiering, 2011; Sepulcre et al., 2012; Buckner, 2013), have undergone massive expansion and an increase in connectivity. These regions also exhibit strong preferences for long-range connectivity (Duncan, 2001; Preuschoft, 2004; Desmurget and Turner, 2010; Sepulcre et al., 2010; Balsters and Ramnani, 2011; Callu et al., 2013) and multimodal information processing (Hikosaka et al., 1999; Lehericy et al., 2005; Poldrack et al., 2005; Mainzer, 2008; Hertel and Brozovich, 2010; Seger and Spiering, 2011; Sepulcre et al., 2012), as expected if they play a critical role in adaptive behavior (Barton and Harvey, 2000; Duncan, 2001; Weaver, 2005; Balsters and Ramnani, 2011; Barton and Venditti, 2013; Callu et al., 2013; Smaers and Soligo, 2013).

## Does the brain work as a computer?

Although metaphors of brain function that invoke computers are often criticized, an analogy with computer-based memory may help to explain the concepts at the core of the “delegation” hypothesis. The difference between the two major systems we identify is similar to the difference between Rapid Access Memory (RAM) and Read-Only Memory (ROM) in a computer. RAM is a flexible and high capacity storage system that is adaptable to multiple functions and can be rapidly modified by user-defined processing decisions, whereas ROM is a rigid, inaccessible system that performs automatic tasks but is outside the control of the system operator. Viewed through this analogy, our hypothesis suggests that the computational problems associated with bipedal locomotion led to the development of a system that catalyzes the “delegation” of behaviors from RAM into ROM, effectively “hard-wiring” rewarded behaviors into memory. By so doing, the brain is able to release computational processing power for delegation to other, more pressing tasks. Importantly, this analogy is about ***function*** and not mechanism: we do not suggest that the computational algorithms used by the human brain are similar to those required for RAM and ROM. Indeed, it is far more likely, based on the precise connectivity of the human brain, that the process of “delegation” from goal-directed to habitual behavior emerges as a function of complex dynamics within neuronal networks (Weaver, 2005; Mainzer, 2008; Balsters et al., 2010; Hertel and Brozovich, 2010; Smaers et al., 2011, 2013).

This analogy also suggests the intriguing possibility that subjective conscious experience is the “user interface” employed by an organism—and that delegation of processing enables us to be aware of (and thus, able to react to) only a small subset of issues (those which benefit from rapid flexible decision-making). In a computer, the user interface offers a simple and accessible model for interacting with the software and hardware, with the complex (and often incomprehensible) language of these systems hidden from the user's view. Such a system allows the apparent mystery of subjective first-person consciousness to be reframed as an organism's individual schematic internal model of its own attention, created by interactions between its own neural systems (Bostan et al., 2010, 2013; Graziano and Kastner, 2011; Graziano, 2013). Similar to the computer analogy, the user interface only affords access to the contents of RAM (i.e. goal-directed processes), keeping the contents of ROM (i.e., habitual responses) hidden deep within the “black box” and inaccessible to consciousness. This analogy also is consistent with the notion that subjective consciousness is not a capacity that divides humans from other animals: instead, it is present across many mammals, birds and even invertebrates (Edelman and Seth, 2009; George and Hawkins, 2009). Importantly, we are not suggesting that the human brain requires a “ghost in the machine” to work effectively, rather that important aspects of conscious experience can be conceptualized as the degree of access that an organism has to its' competing memory systems. Nonetheless, the systems that the brain relies on to mediate our vast behavioral repertoire have similarities with (as well as differences from) the memory systems of a computer. Recognizing those analogies may help us to understand the far more complex mechanisms underlying human cognition. Indeed, a particular strength of the proposed model is that increasingly complex behaviors can *emerge* from this system without the need for conscious control, as behaviors are delegated to automaticity through repetition, and via minimization of reinforcement-based error signals.

## Testable predictions

How can we test the predictions of this model? Studies on brain function have clarified the manner in which actions can shift from goal-directed to habitual behavior (Poldrack et al., 2005), but our hypothesis suggests a more prominent role for the cerebellum (particularly as behaviors become more habitual and rigid) than has been suggested by previous authors (Yin and Knowlton, 2006; Balleine and Delgado, 2007). We could test that prediction by assessing the brain during the course of learning, using a range of complementary neuroimaging techniques. In addition, we know of no direct evidence that the shifting process occurs at a more rapid rate or more efficiently in humans than in non-human species; however such a divergence is an explicit prediction of the “delegation” hypothesis. If suitably framed, comparative learning studies in human and non-human primates could specifically test that prediction. Our hypothesis also could be tested by a detailed analysis of the brains of extant animals (especially primates) as well as fossil braincases from proto-hominids, to determine whether brain morphology covaries with the acquisition of bipedality, and/or the ability of different taxa to “delegate” rewarded behaviors into automatic functions (Holloway et al., 2004). On a genetic level, we expect that changes in delegation ability should be mirrored by disproportionate alterations in both coding and non-coding genetic activity in the brain (Mattick and Mehler, 2008), particularly in the cortical, cerebellar and basal ganglia circuitry that is likely to be important for the delegation of behavior to automaticity (Figure 1). Although studies on the genetic basis of human behavior are in their infancy, early results on the neural distribution of non-coding regions in the genome support this general notion (Mattick and Mehler, 2008).

We could also test the hypothesis by exploring variation in behavior within humans. Notably, the neuroanatomical relationships described above could be explored in neurobiological disorders—specifically, those in which individuals suffer from impairments in automaticity. Preliminary evidence suggests, for example, that patients with Parkinson's disease (which is due to impairments in the circuitry of the basal ganglia Dayan and Balleine, 2002; Poldrack et al., 2005; Obeso et al., 2008) exhibit reduced development and recruitment of habitual behaviors (Poldrack et al., 2005). In addition, patients with cerebellar lesions also show deficits in the execution of automatic behaviors(Lang and Bastian, 2002; Callu et al., 2013). Other neuropsychiatric disorders, such as obsessive-compulsive disorder (Gillan et al., 2011, 2013; Robbins et al., 2012) and post-traumatic stress disorder (Arnsten, 2009), may also be amenable to reconceptualisation through the framework of the “delegation” hypothesis. Indeed, there is now evidence that patients with schizophrenia have impaired habitual performance on cognitive tasks (Horan et al., 2008; Wagshal et al., 2012), perhaps explaining why patients with the disorder are impaired across multiple cognitive domains (Fornito et al., 2011).

Our hypothesis also predicts significant phenotypic variation among healthy patients in the ability to delegate behavior to automaticity. Specifically, we expect that the ability to delegate behavior to automaticity may correlate with high performance on the tests commonly used to measure general intellectual expertise. Indeed, if variation in the ability to automatize behavior does indeed underlie people's differential performance in cognitive tests, we would predict commonalities in the brain-function mechanisms that confer expertise across multi-dimensional domains, such as Art, Science and Music. If experts in all these fields utilize similar brain mechanisms to achieve mastery of their chosen task, which it appears as though they do (Beilock and Lyons, 2012; Bilalić et al., 2012), the implications for performance improvement (and remediation of poor performance) are clear. Future studies using functional neuroimaging could usefully investigate the precise spatiotemporal dynamics that underlie the conversion from goal-directed to automatic behaviors, as well as the relative role played by the cortex, basal ganglia and cerebellum in the acquisition, execution and maintenance of adaptive behavior.

Finally, our hypothesis predicts a tradeoff: the automatization of behavior (1) should reduce an individual's ability to consciously attend to habitual function; and (2) enhance their ability to react rapidly and effectively to novel (unexpected) inputs. For example, habitual behaviors should be much less accessible to conscious exploration than those behaviors that require flexible, “on-line” control. Interestingly, the breakdown of this relationship between conscious access and automaticity may explain the well-documented phenomenon (known in the sporting world as the “yips”) whereby an individual who inappropriately accesses a usually habitual activity at a conscious level, can thereby commit uncharacteristic errors (Beilock and Gray, 2007).

## Conclusion

In summary, the massive neurocomputational challenges posed by the onset of bipedality may have been the driving force behind the rapid expansion of human cognitive capacity. Specifically, the ability to rapidly “delegate” well-learned behaviors into subconscious processes liberated higher neural centers to be available for flexible, “on-line” processing of fitness-relevant stimuli. Our ideas suggest several testable predictions and may clarify not only how human cognitive systems differ from those of other species, but also how the human brain works both in health and disease.

### Conflict of interest statement

The authors declare that the research was conducted in the absence of any commercial or financial relationships that could be construed as a potential conflict of interest.
